# Finite Element Analysis of Upper Limb Splint Designs and Materials for 3D Printing

**DOI:** 10.3390/polym15142993

**Published:** 2023-07-10

**Authors:** Syed Hammad Mian, Usama Umer, Khaja Moiduddin, Hisham Alkhalefah

**Affiliations:** 1 Advanced Manufacturing Institute, King Saud University, Riyadh 11421, Saudi Arabia; 2 King Salman Center for Disability Research, Riyadh 11614, Saudi Arabia

**Keywords:** finite element simulation, upper limb splint, polymers, customization, 3D printing, perforated designs, topology optimization, reverse engineering

## Abstract

Three-dimensional (3D) printed splints must be lightweight and adequately ventilated to maximize the patient’s convenience while maintaining requisite strength. The ensuing loss of strength has a substantial impact on the transformation of a solid splint model into a perforated or porous model. Thus, two methods for making perforations—standard approach and topological optimization—are investigated in this study. The objective of this research is to ascertain the impact of different perforation shapes and their distribution as well as topology optimization on the customized splint model. The solid splint models made of various materials have been transformed into porous designs to evaluate their strength by utilizing Finite Element (FE) simulation. This study will have a substantial effect on the designing concept for medical devices as well as other industries such as automobiles and aerospace. The novelty of the research refers to creating the perforations as well as applying topology optimization and 3D printing in practice. According to the comparison of the various materials, PLA had the least amount of deformation and the highest safety factor for all loading directions. Additionally, it was shown that all perforation shapes behave similarly, implying that the perforation shape’s effect is not notably pronounced. However, square perforations seemed to perform the best out of all the perforation shape types. It was also obvious that the topology-optimized hand splint outperformed that with square perforations. The topology-optimized hand splint weighs 26% less than the solid splint, whereas the square-perforated hand splint weighs roughly 12% less. Nevertheless, the user must choose which strategy (standard perforations or topology optimization) to employ based on the available tools and prerequisites.

## 1. Introduction

A resting splint or a static orthopedic brace is worn to prevent movement in the upper limbs, such as the wrist, fingers, and thumb during rehabilitation for motor dysfunctions. The splint is intended to facilitate healing by providing rest for the affected joint(s), preventing and treating abnormalities, increasing the joint’s range of motion, and enhancing overall hand and upper limb function [1]. However, despite being strongly advised by experts in the field, the World Health Organization reports that in about 35.4% of cases, rehabilitation devices are used inconsistently or rejected because they cause the patient discomfort or the expert cannot modify the device to the patient’s needs [2]. 

Splint creation, application, and design are both scientific and artistic endeavors. Splints that are improperly made and administered have the potential to do much more harm than help. It is well accepted that a patient’s failure to comply with the prescribed medical or therapeutic regimen can lessen the advantages of therapy, slow the patient’s progress toward recovery, raise the patient’s risk of becoming disabled, and lead to erroneous conclusions about the treatment’s effectiveness. According to research conducted by Safaz et al. [3], the primary reason for patients abandoning the use of orthoses is pain and discomfort. In addition, the restricted aesthetic modification and the lack of fresh designs are further factors that deter the adoption of orthoses [4]. Casting and splinting with plaster or fiberglass are the traditional methods for stabilizing the hands and wrists [5]. Traditional splinting starts with putting a deep layer of soft padding next to the skin, a middle layer of stiff fiberglass or plaster of Paris, and a top layer of compression wrap to hold it all together. The orthopedic surgeon checks the clinical condition and, finally, X-rays are employed to look at the cast and decide how stable it is. If the splint is too tight, it may need to be loosened or adjusted, and the casting process may need to be carried out again. Several research studies have stated the problems associated with this technique which include the heaviness of the cast, sweat, difficulty in staying dry, difficulty in adjusting after placement, and skin problems [6,7]. This drives the exploration for non-intrusive substitutes for obtaining anatomical configurations using reverse engineering (RE). The initial phase of an RE methodology involves the acquisition of hand anatomy, which can subsequently be utilized to attain significant levels of customization in orthotic devices. The methodology typically comprises three primary phases, as outlined by other research studies [8,9]. The process involves three main steps: firstly, the anatomical parts are scanned utilizing a three-dimensional (3D) scanner; secondly, the acquired geometry is processed and the splint model is designed through the use of computer-aided design (CAD) software; and finally, the physical splint is fabricated using additive manufacturing (AM) technologies. 

Optical scanning has been identified as the most appropriate 3D data acquisition technology for hand data acquisition, based on criteria such as accuracy, resolution, patient safety, cost, speed, and efficiency [10,11]. The scanning duration, as well as practicality, mobility, and adaptability, are the most important scanner criteria in orthotics [12]. Likewise, AM enables the comparatively simple realization of very complex digitally produced models with little or no human intervention in the manufacturing process or in-depth material expertise [13]. With the help of an organized, layer-by-layer build process, objects can be constructed in complicated configurations that would otherwise be impossible to create using conventional manufacturing techniques [14]. Many of the issues related to conventional splinting, such as poor aesthetics and inadequate ventilation, might be alleviated by employing AM. This technology’s ability to instantly replicate the human form utilizing optical surface scanning [15] and medical imaging [16] datasets has further enhanced its potential. The design and production of orthotic devices that conform precisely to the anatomical features of patients can enhance their quality of life by providing personalized shape, performance, function, and aesthetics [11]. The process of creating custom handmade orthoses through manual fabrication using low-temperature thermoplastic material, as recommended by the International Committee of the Red Cross, can be a challenging, laborious, and time-intensive task [13]. Additionally, this method carries the potential risk of minor skin burns or irritations owing to direct exposure to heated material [17]. The additively manufactured splints take 3 h in the fabrication of personalized orthoses when compared to 3 to 4 min for a plaster cast, but the plaster cast takes approximately a whole day (24 h) to become fully set onto the hand, thus demonstrating a big reduction in time with 3D printing.

Several research and case reports have presented various design strategies for 3D splints and hand surgery assistance devices [18]. The 3D-printed cortex cast was one of the first splints fabricated using a lattice design to stabilize and immobilize the bone fractures as well as provide air circulation [19]. Similarly, ActivArmor utilized AM technology to produce commercial personalized orthoses in the United States of America [20]. Paterson et al. [21], in their study, proposed a low-temperature thermoplastic perforated splint to reduce the swelling protrusion and increase the material strength. A variety of AM prototypes, including single and multiple material splints, have also been investigated by the designers to illustrate the possibilities of AM for bespoke splint production [22]. However, these methodologies provided little possibility for the recording of practitioners’ preferences for fit and performance, and they did not suggest a method for incorporating alternative lattice forms to accommodate specific patient preferences in styles [23,24]. Although a few studies have discussed the fabrication of lattice and perforated splints, none have described the detailed process flow for its creation from 3D-scanned data to AM fabrication. 

The patient-specific splints created from 3D scans offer wearing comfort and an elegant look. The mesh-like construction provides great ventilation but sometimes the structure is too weak to support the limb. In addition, a low-intensity mechanical impact can readily shatter the webbed beam. Furthermore, due to the narrow connecting bars, the webbed design is prone to breakage and cracking. These sophisticated designs are still being evaluated technically, with no clinical applicability or approval. Hence, FE analysis is one of the best tools to verify the design and the engineering strength of the proposed design [25]. FE analysis is described as the process of understanding, analyzing, and predicting the behavior of an item, component, assembly, or structure under various physical situations using mathematical calculations, models, and simulations. Lin et al. [26], used FE analysis to assess the mechanical performance of a 3D-printed splint and to detect the potential risk of structural collapse owing to concentrated stresses in their investigation. Chen et al. [27], conducted a study wherein they presented an integrated FE model that incorporated a forearm and a 3D-printed cast; the study demonstrated that the use of a patient-specific 3D-printed cast with a wearer-friendly design was beneficial in maintaining the alignment of forearm fractures, enhancing patient comfort and reducing the likelihood of complications.

AM or 3D printing systems are a viable alternative methods for manufacturing these intricate orthopedic devices. They have opened up fresh possibilities for producing intricate shapes with undercuts or cavities and for minimizing waste [28]. The porous/perforated designs, or the topology optimization process and 3D printing, when integrated, allow professionals endless options and expand the range of potential solutions for the development and production procedure. The orthoses made by AM can give patients individualized rehabilitative tools, leading to a more appealing design, reduced weight, and reduced volume, as well as better airflow, all of which have a good effect on the patient’s usage of the product [2,29]. Additionally, it is critical to perform initial analyses on orthoses to estimate their strength and to confirm that the right material is being used and the perforations are suitable before producing on AM [30]. 

As observed in the literature review, a large number of orthoses have rather high procurement costs because a considerable amount of material is needed in their production [2]. The price of obtaining an orthosis can be a significant barrier to accessing recuperation facilities, such as hand splints. Moreover, the research carried out by Safaz et al. [3], found that the strain caused by the orthosis is the main reason for its dislike among the users. According to Lau [31], the orthosis’s weight is the main cause of the patient’s distress and unhappiness. The restricted aesthetic customization of the orthosis and the lack of fresh designs that could be made available to the user are additional factors that deter its use [4,32]. The application of perforated orthoses should be advocated to address the aforementioned problems with orthopedic equipment. There are two common approaches for making pores or removing material from orthoses: perforations with standard shapes, and topology optimization, the latter of which is carried out using the software’s built-in program. Therefore, it is crucial to look into the two strategies to comprehend their outcomes and implications. Another challenge related to these perforated orthoses is their production owing to their complexity. 

The key objectives of this research are to investigate perforation shapes and their distribution, to examine topology optimization for an upper limb orthosis, to compare various materials, and to assess the viability of 3D printing for a perforated orthosis. To calculate Von Mises stresses and safety factors, FE numerical analysis was performed for both perforated and topology-optimized splints, and the resulting orthosis was printed using fused deposit modeling (FDM). The goal was to make the splint lighter while preserving its appropriate level of strength compared to the original solid body. The fundamental challenge with FE simulation is to precisely reproduce the anatomy in any portion of the human body. Thus, this study also devised a methodology that incorporates a RE design approach based on data collection through laser scanning and splint design from the collected point cloud data.

The manuscript is organized as follows. The problem, the research gap, and the aims are introduced in the Section 1. Materials and Methods, which is Section 2, provides an explanation of the RE methodology, FE numerical analysis, topology optimization, and 3D printing fabrication. Section 3 is the Results and Discussion, which analyzes the various orthoses’ designs as well as fabrication materials and discusses the study’s findings. The work and its outcomes are finally summarized in Section 4.

## 2. Materials and Methods

**Figure 1:**
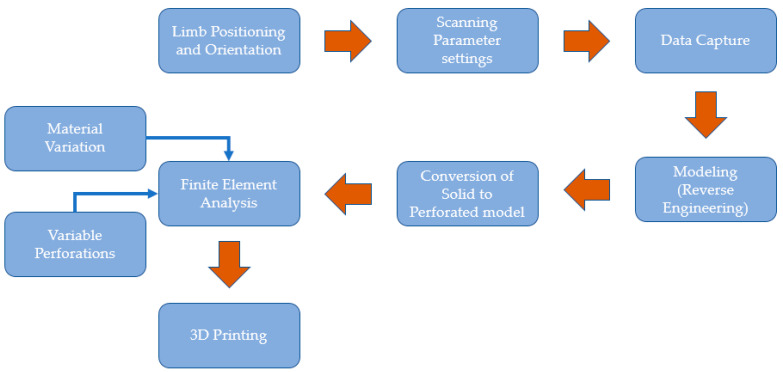
Flow chart summarizing the methodology.

The methodology developed in this study (Figure 1) includes data collection, an RE design approach (or modeling technique), a numerical analysis process, and 3D printing. This study uses FE to assess several wrist splint designs based on perforation shapes, distributions, and materials. This section also includes technical directives to streamline the procedures associated with the established methodology. Various steps and techniques used during the development of the proposed approach for splint design and manufacturing using modeling tools are presented to address problems that arise at different phases.

### 2.1. Data Acquisition

**Figure 2:**
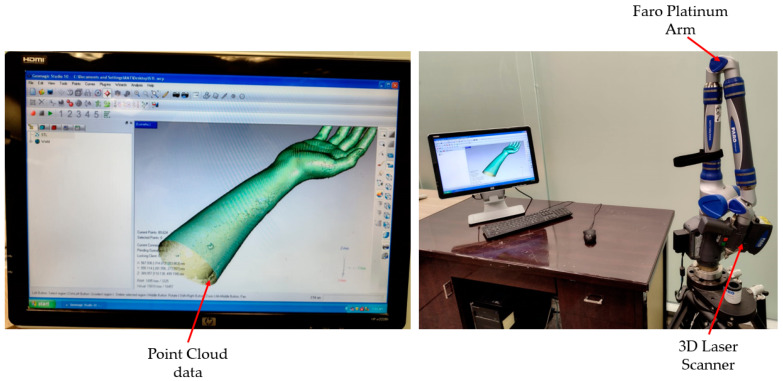
Scanning system for data capturing and gathered point cloud data.

The 3D information gathered from the laser scanning equipment was inputted into the hand splint modeling process. The research team’s fit volunteer’s hand was leveraged to capture data. The acquisition of physical information on the participant’s limb constitutes one of the methodology’s essential steps. As shown in Figure 2, the scanning was performed with the Faro Platinum arm (FARO, Lake Mary, FL, USA). The individual’s Standard Tessellation Language (STL) file was obtained through 3D scanning to create the tailored splint utilizing the RE. 

An individual (or limb) is digitally scanned using a laser in 3D space to obtain as large a surface as achievable to improve design freedom. To posture the individual comfortably, the upper limb (or hand) is elongated as far as it can be extended without becoming stiff or bothersome.

The best scanning outcomes can be achieved by carefully establishing the scanner’s settings [33]. For example, slower scanning rates could result in inaccuracies from inadvertent motions, and quicker scanning rates could result in the scanner missing some areas. Additionally, it is essential to adjust the sensor’s acquisition parameters to account for surface color, unevenness, and ambient lighting. For the best scanning, keeping a safe distance from the surface is also required. The proper definition of several important scanner variables, including the scan rate, scan density, exposure time, noise threshold, data format, etc., is necessary for effective scanning. The scan rate is maintained at 1/1, which is the default value (or the normal rate) recommended by the manufacturer for the highest degree of precision and is defined as the number of scan lines per second. Thirty scan lines are collected per second, according to the 1/1 scan rate setting [34]. The same goes for the scan density, which is set to 1/1 and determines how many points are present on each scan line. The scan density of 1/1 implies that all points (i.e., 640 points) on the laser line are captured in the sensor. The scanning equipment automatically determines the laser exposure settings based on the shape and reflectivity of the surface being scanned. The noise threshold, which gauges the amount of noise in the data, is automatically chosen by the software based on the surface and the surrounding conditions. The amount of light or the power of each pixel sent onto the surface by the laser line scanner is measured depending on the ambient condition. The “noise” or “chatter” applies to all data with an intensity that is lower than the noise threshold value. The ordered data are represented by a point cloud with a consistent density and points structured in linear rows and columns. The unorganized (or raw) data are a point cloud with a random distribution of points. With raw data settings, the points are captured at each location where the laser line falls on the object. The raw data approach is used because it gives higher precision and records every feature of the scanned part. The above-mentioned parameters have to be set before the scanning process begins. 

**Figure 3:**
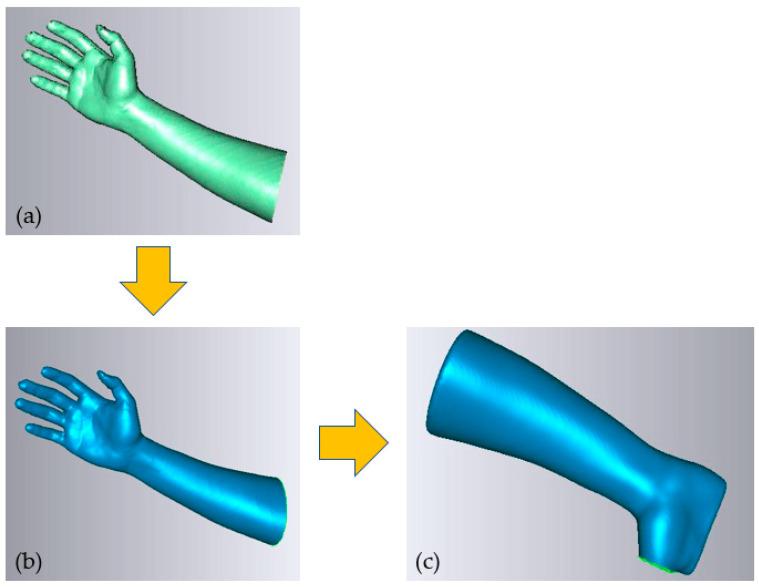
Scanning outcome (**a**) Point cloud (**b**) Converted mesh (**c**) Final mesh for splint modeling.

The output of the scanning process was in the form of point cloud data (Figure 3a), which were then processed and converted into a polygon model, as seen in Figure 3b. The polygon model included error components, such as incorrectly captured surfaces or faces omitted from crucial locations. To increase the effectiveness of the splint design process, the polygon model was cleaned, optimized, and prepared for the following design and modeling steps. The processing operations performed included noise and outlier expulsion, erroneous facet repairs, mesh smoothing and shaping, hole filling, face flaw correction, etc. The resultant polygon model in Figure 3c was then saved in STL format to model and produce 3D splints.

### 2.2. Design and Modeling

**Figure 4:**
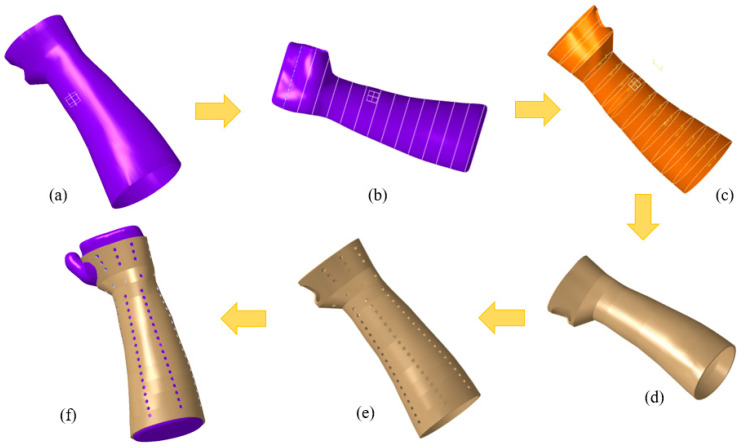
Modeling of the hand splint (**a**) Mesh of the hand (STL file); (**b**) Extraction of curves on the mesh; (**c**) Surface generated on curves; (**d**) Translation of surface to part model; (**e**) Conversion of the solid model into a perforated model; (**f**) Splint placed on the mesh.

The next step was to create a unique hand splint model by applying the mesh form (STL file) shown in Figure 4a. The deployment of interpolation spline curves serves as the starting point for creating a splint that is specific to the individual’s hand anatomy [35]. The modeling process was accomplished with Catia V5 (R20, Dassault Systèmes, Vélizy-Villacoublay, France) by combining multiple phases, such as the Digitized Shape Editor, Generative Shape Design, and Quick Surface Reconstruction. The reported methodology is ubiquitous and can be applied to either an upper or lower limb. In the Digitized Shape Editor, as seen in Figure 4b, the spline curves enclosing the hand’s surface were defined as the initial step in the modeling process. The Planar Section option was chosen for this operation. It cuts a mesh or a cloud of points using planes to derive curves. Wherever the curves are separated from one another, the shape of the curves is interpolated from the existing surrounding nodes, culminating in a spline curve that seamlessly spans all of the required points. To achieve this, the Connect Curve feature in Generative Shape Design was implemented to create a bridging curve that connects two curves. Finally, surface patching was made possible by the generated spline curves. The process of producing surfaces used the Quick Surface Reconstruction module of the CATIA V5 software. Figure 4c shows how the multi-section surface approach was used to create the surface patch in accordance with the curvature of the spline curve. The split function in the Generative Shape Design was then applied to make the hole where the thumb is supposed to be. The process was completed when the splint surface model was transformed into the part model, as seen in Figure 4d. Once the splint model had been created, it could be customized with various perforation shapes and patterns for ventilation and weight reduction. Figure 4e depicts, as an illustration, a splint with circular perforations. As shown in Figure 4f, the splint was meticulously set on the hand mesh or blueprint. 

**Figure 5:**
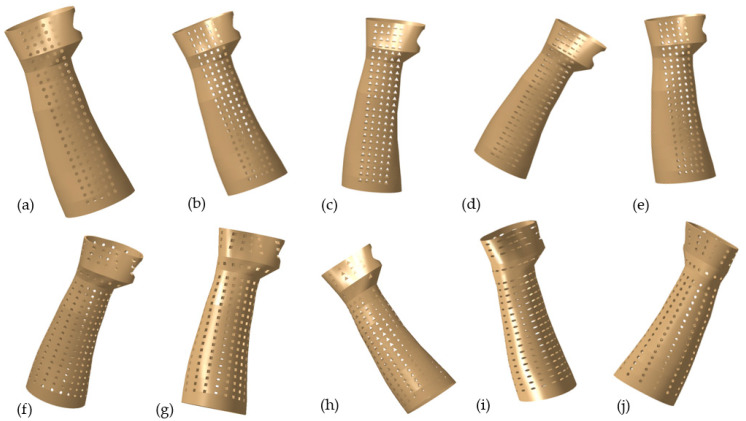
Splint designs—Linear/Packed (**a**) Circular; (**b**) Square; (**c**) Triangular; (**d**) Elliptical; (**e**) Hexagonal; Round/Scattered, 30°(12) (**f**) Circular; (**g**) Square; (**h**) Triangular; (**i**) Elliptical; (**j**) Hexagonal.

The perforations are constructed using a variety of shapes, including circular, square, triangular, elliptical, and hexagonal. In this particular work, the perforations’ distribution and shapes were both altered. The details of various designs, such as linear and round, are depicted in Table 1 and the splints were established with a 3 mm thickness. For example, Figure 5a illustrates the design with circular perforations and a linear/packed pattern (Design 1). Here, in Design 1, the perforations are dispersed linearly as well as packed in a particular area on the splint. Similarly, the perforations are dispersed evenly in a round and scattered pattern (with 30°/12) across the splint according to Design 3 (see Figure 5f). A 30°/12 denotes that there are twelve perforations at each level, all of which are at a 30° angle. Other designs and perforations in Figure 5b–j can be interpreted in a similar fashion. The dimensions used to create the various perforation forms were selected so that they all result in the same area (see Table 1).

### 2.3. Finite Element Analysis

This investigation focuses on comparing several wrist splint designs and materials through Finite Element (FE) analysis. The FE performed in this research was developed based on the methods presented by Li and Tanaka [36]. The various designs of splints that include different perforation shapes (circular, square, triangle, ellipse, and hexagon), variable perforation distribution, and multiple materials, including Polylactic Acid (PLA), Acrylonitrile Butadiene Styrene (ABS), Thermoplastic Polyurethane (TPU), Polypropylene (PP), and Polyethylene terephthalate glycol (PETG), were taken into consideration in the current work. The material properties incorporated in the FE model as depicted in Table 2 were acquired through the literature and online resources.

The software employed for the FE analysis was Abaqus from Dassault Systems (Dassault Systems, Vélizy-Villacoublay, France). The designed splint was imported into Abaqus via the Initial Graphics Exchange Specification (IGES) file format. The FE model was generated and resulted in 25,184 node points and 37,227 linear four-node tetrahedral elements (C3D4) for the splint. A mesh sensitivity study was also carried out before finalizing the mesh configuration. Further mesh refinement (e.g., 10 times more elements) was found to result in a threefold increase in computational time with a less than 1% difference in the stress and displacement values.

General static simulation was used in FE analysis using ABAQUS/STANDARD solver to assess the stiffness of various splint designs applying known forces. The splint model’s circular corners and screw slots were eliminated to make simulation computations easier. Considering the load-displacement scenarios, linear elastic behavior was assumed. Nonlinear effects such as large displacements and material nonlinearity were ignored. In addition, material was assumed to be homogenous and isotropic, i.e., material properties did not vary spatially and had the same properties in all directions at a particular point. The material was defined using Hooke’s law, i.e., two parameters including Young’s Modulus (*E*) and Poisson ratio (*ν*) were needed. The proximal edge was designated as the fixed base when the boundary conditions (BCs) were established. The different analyses were performed by changing the material, perforation shapes, and density. 

**Figure 6:**
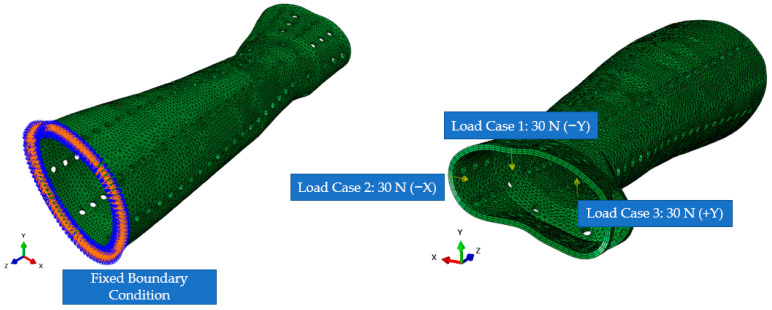
FE mesh, BCs, and Load cases (or directions).

A structural input load of 30 N was imposed on the distal edge of the splint and patterned area along three directions independently to imitate possible traumas and pressures that could accidentally happen through the recuperation time [36]. The FE mesh, BCs, and load directions are shown in Figure 6.

The displacements and stresses were investigated using a number of FE analysis simulations. These simulations explore the effectiveness of the various materials as well as the impact of the various perforation shapes and their distribution on the splint. Slight deformations were considered in all simulations, meaning the materials remain in their elastic region of the stress–strain curve. The simulations were carried out using a static, all-inclusive procedure that took advantage of ABAQUS/STANDARD’s implicit solver. These analyses calculated the displacements and the Von Mises stresses. All simulations were performed on a computer running Windows 10, 64 bits, an Octa-core Xeon processor operating at 3.7 GHz, 16 GB of RAM, and each simulation required roughly one minute.

### 2.4. Topology Optimization

The hand splint was also assessed through topology optimization, which is an automated process that employs the same BCs as the FE analysis of perforated models stated before. When it comes to engineering design, the topology optimization process, which relies on mathematical algorithms, greatly simplifies the task of a designer by recommending alternative design options. Considering a design space and a set of constraints (such as loads and BCs), topology optimization is an example of the computational approach that makes it possible to determine the best material distribution within that space [51,52]. The overall weight of the intended product can be significantly reduced while still retaining the appropriate strength and rigidity with the application of topology optimization in the design process [53]. 

Utilizing the proper splint thickness, the model’s topology was optimized in the Abaqus Topology Optimization Module. Condition-based topology optimization was utilized in this study. This Abaqus application implements an algorithm that computes the most efficient surface design after implementing a set of constraints meant to remove material. The topology optimization has a standard formulation to reduce the material’s excess volume while still adhering to the pre-defined limits. 

Abaqus software uses TOSCA STRUCTURAL OPTIMIZATION SOLVER in the background, and thus the topology optimization was based on the Solid Isotropic Material with Penalty (SIMP) method. By discretizing the domain into an FE mesh, TOSCA calculates material properties for each element. The TOSCA (SIMP) algorithm alters the material distribution to optimize the user-defined objective under given constraints. In the SIMP method, a pseudo material density is the design variable, and hence it is often called the density method as well. As compliance is given by U^T^KU, the condition-based optimization problem can be given as:

$$\mathrm{Min}:U^{T}KU\;\mathrm{Constraints}:\;\sum_{e=1}^{n}v_{e}\rho_{e}\leq V\;\rho_{e}=\{0,1\}$$

where K is the global stiffness matrix, U is the global displacement vector, $v_{e}$ is the element volume, $\rho_{e}$ is the relative element density and *n* is the number of elements in the optimization problem. As stated above, the design variable can take only integer values, i.e., 0 or 1. This method is computationally expensive so the integer value problem is related to:

$$0\leq \rho_{e}\leq 1$$

This changes the integer value problem into a continuous design variable problem. The SIMP method is used to avoid intermediate density elements in the final solution by scaling the stiffness of the intermediate density elements using a penalty factor *p* and it is given by:

$$K_{SIMP}=\sum_{e=1}^{n}[\rho_{min}+(1-\rho_{min})\rho_{e}^{p}]K_{e}$$

where $K_{e}$ is the element stiffness matrix, $\rho_{min}$ is the minimum relative density, $\rho_{e}^{p}$ is the element’s relative density, and *p* is the penalty factor.

The topology optimization process has a few parameters to set up before optimization begins. The topology optimization process was set up to maximize the stiffness of the component (or minimize the compliance) when reducing its mass with a final mass percentage target set at 70% of the existing splint’s body. All parameters are shown in Table 3.

The volume target specified the amount of material to keep. The volume constraint was set as a percentage (70%) of the total volume of the design space. Element size dictates the quality of the optimization result. In general, the smaller the element size, the more accurate the result but the longer the time required to perform a computational process. Depending on the computational power and limited time for this study, the element size was set at the value of 3.4 mm. 

**Figure 7:**
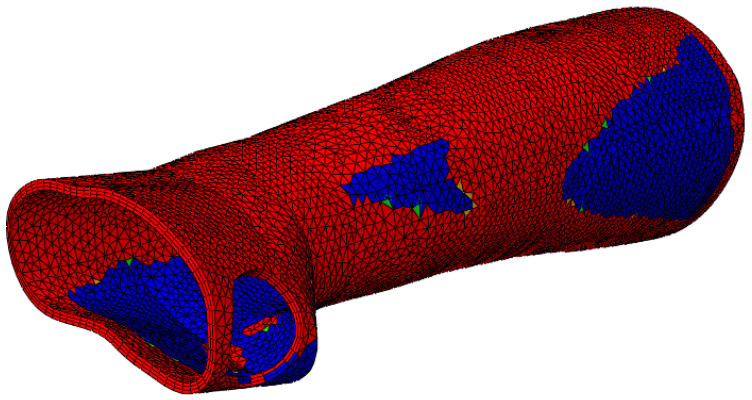
Classification of the material by topology optimization to be preserved and removed.

Before the topology optimization started, it was important to specify the places that must not be optimized (non-design space). Thus, in this study, for the topology optimization of the model, a 3 mm thick orthosis with the same BCs as the initial FE analysis of the perforated models was considered. The blue/green portions are those that the program recommends for material removal (soft elements), while the red regions are those that are indicated for surface retention (hard elements) (Figure 7). After topology optimization, the newly generated model was further put to examination using FE analysis. The loads, BCs, etc., which were needed to solve the optimization process are the same as those shown in Figure 6.

### 2.5. Fabrication

**Figure 8:**
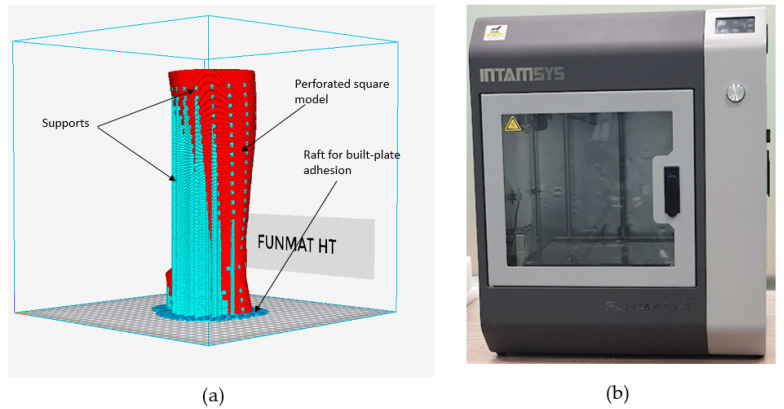
(**a**) INTAMSUITE slicing software; (**b**) Funmat HT 3D Printer.

The STL file’s errors must first be fixed before the modeled splint can be produced. The triangles (in the triangulated polygon model) that are overlapping, intersecting, have twisted edges, and other issues can all be fixed with Magics^®^. The goal of this stage is to prepare the STL file for manufacture by identifying the appropriate location and alignment as well as building support structures wherever they are required. The proper supports are necessary to develop the prototype precisely while avoiding any irregularities. Additionally, they are imperative to prevent warping and for efficient heat transmission. In this investigation, the zigzag support pattern was employed as the support structure due to its expeditious printing speed, optimal equilibrium, and having the simplest removal process, as well as its efficacy to preserve the integrity of the 3D print. As demonstrated in Figure 8a, the INTAMSUITE slicing software (Version 3.6.2, INTAMSYS Technology Co., Ltd., Shanghai, China) was employed to slice, position, and create supports. The INTAMSYS (Intelligent Additive Manufacturing Systems) FUNMAT HT 3D printer (INTAMSYS Technology Co., Ltd., Shanghai, China) was deployed to produce the personalized hand splint (Figure 8b) [54].

The STL splint file was first converted into G-code through the embedded slicing program INTAMSUITE (Shanghai, China), and then it was loaded onto an SD card and sent to the Funmat HT 3D printer. The printer uses FDM technology, which has the advantage of using less energy, producing more material, and providing greater tensile strength [55]. FDM is among the most prevalent and basic of AM processes [56]. The machine includes dual nozzle extruders, one for normal temperature engineering polymers such as PLA, ABS, PP, TPU, etc., and another for advanced elevated temperature materials such as polyetheretherketone (PEEK) [57]. The extruder nozzle is adjustable depending on the choice of printing material. The manufacture of 3D-printed components depends on numerous factors. Therefore, it is essential to choose the appropriate conditions when producing any part. The process parameters used in the splint manufacture are listed in Table 4. The printer includes a building enclosure that is capable of reaching temperatures as much as 90 °C and a thermal construction plate made of high borosilicate glass that can tolerate temperatures close to 160 °C. The infill density of the slicing software was fixed at 100% to produce a precise and long-lasting structure.

**Figure 9:**
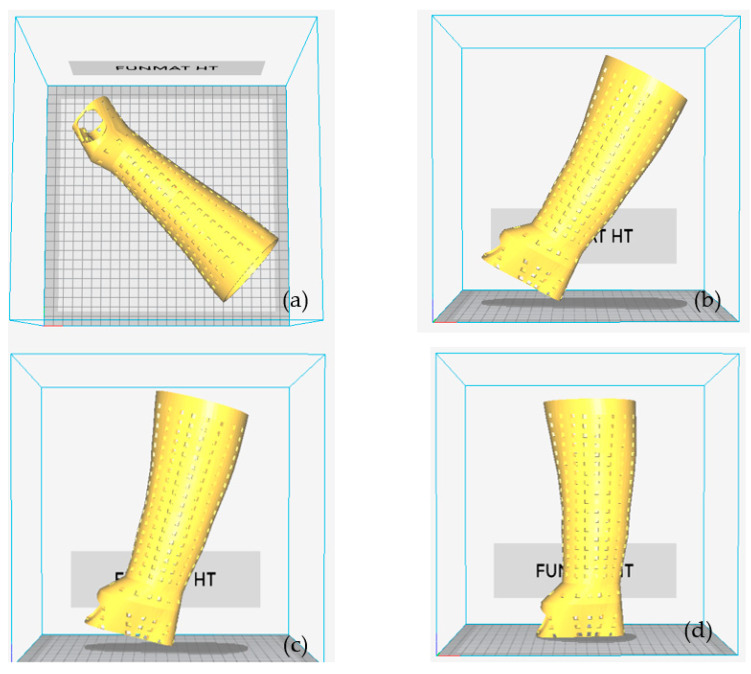
3D printing orientation using INTAMSUITE software: (**a**) orientation 1 (0°), (**b**) orientation 2 (60°) (**c**) orientation 3 (75°) (**d**) orientation 4 (90°).

The component’s orientation affects a number of variables, including the overall amount of material used and the build time. These variables also have an impact on the part-building cost. The total amount of material used, as well as the build time, are both influenced by the part’s geometry and the volume of supports needed. Parts must be positioned correctly on the build platform to make the best use of available space and print time. To discover the proper print orientation on the build platform, an INTAMSUITE was used to loop through all four of the possible orientations indicated in Figure 9. The orientation of the splint represents the angle between the build table and the splint. The orientation is 0° when the splint is flat on the build table. The splint was positioned diagonally on the build table to fit within the building envelope (Figure 9a). It denotes orientation 2, 3, and 4 when the splint is turned 60°, 75°, and 90°, respectively, counterclockwise on the building table (Figure 9b–d). The objective of this task was to evaluate factors, particularly the build time and overall material use for various orientations. The orientation with the best results was chosen for the following FDM process. 

The post-processing of the 3D-printed splint, which involves peeling and removing the support layers, was carried out after the hand splint had been produced. The wall of the support structure was weakened so that fracturing was easy. Protective gloves were worn, as cutting and gripping pliers were used to reach the underside of the supports and slowly bend them upwards to remove them.

## 3. Results and Discussion

The purpose of this discussion is to identify the suitable design and material for minimizing Von Mises stresses and weight. When seeking to estimate the strength of a specific design built of a certain material, the Von Mises stress criteria must be applied. It implies that the specific structure would collapse under the specified loading conditions if the Von Mises stress exceeds the material’s yield strength. Von Mises stress is of the utmost importance in the design of structures because it indicates the total magnitude of all stress components (tensile, compression, and shear) at any given point. This is very helpful for anticipating the modes of failure in engineering designs and figuring out whether a component or structure is strong enough to bear predicted loads. Additionally, Von Mises stress can estimate a component’s safety factor, which aids in determining whether or not additional load can be incorporated into the design. The ratio of the material’s strength to the component’s maximum stress is known as the safety factor or factor of safety. The material would fail under the prevailing loading conditions if the factor of safety is less than 1. It is undoubtedly possible to build structures that can endure extreme loads by understanding Von Mises’s stress and how it relates to the material’s yield strength. 

It would require about three hundred FE analyses to consider five distinct materials, twenty different designs in addition to a topology-optimized structure, and three loading directions. This would need a lot of work, a lot of time, and more expensive calculations. Therefore, rather than comparing all of the design sets for all materials, it was decided to evaluate the different materials based on only two design sets to reduce the computational cost. Finally, the material that performed well in the two design sets would be taken into consideration for the subsequent studies, which would also include three additional design sets. A comparative analysis of different materials was conducted based on deformation and the safety factor under the specified applied loading conditions. The material with the lowest deformation and highest safety factor will be considered for future evaluation.

**Figure 10:**
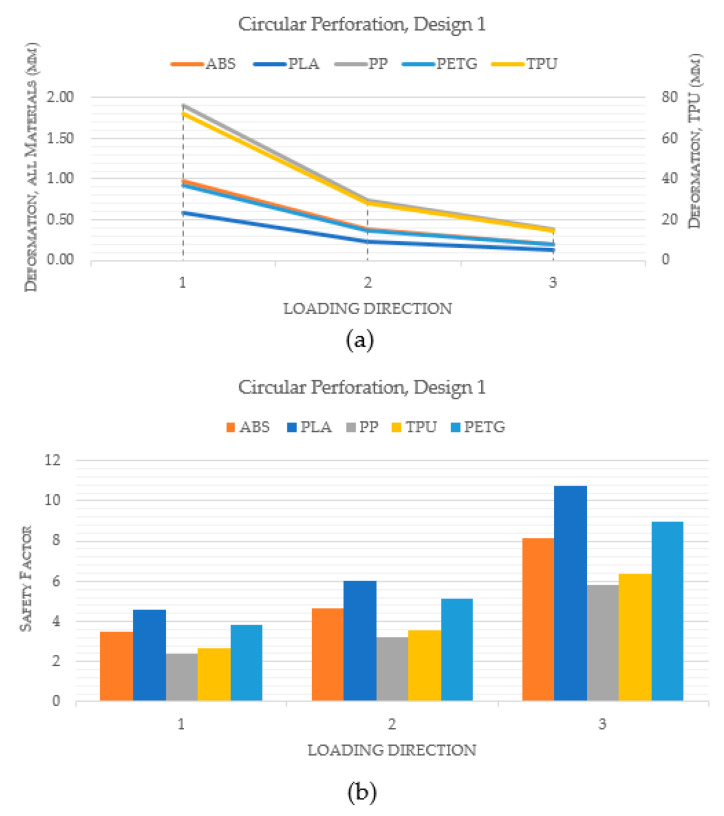
Comparison of different materials with circular perforations and Design 1 (**a**) Deformation; (**b**) Safety factor.

**Figure 11:**
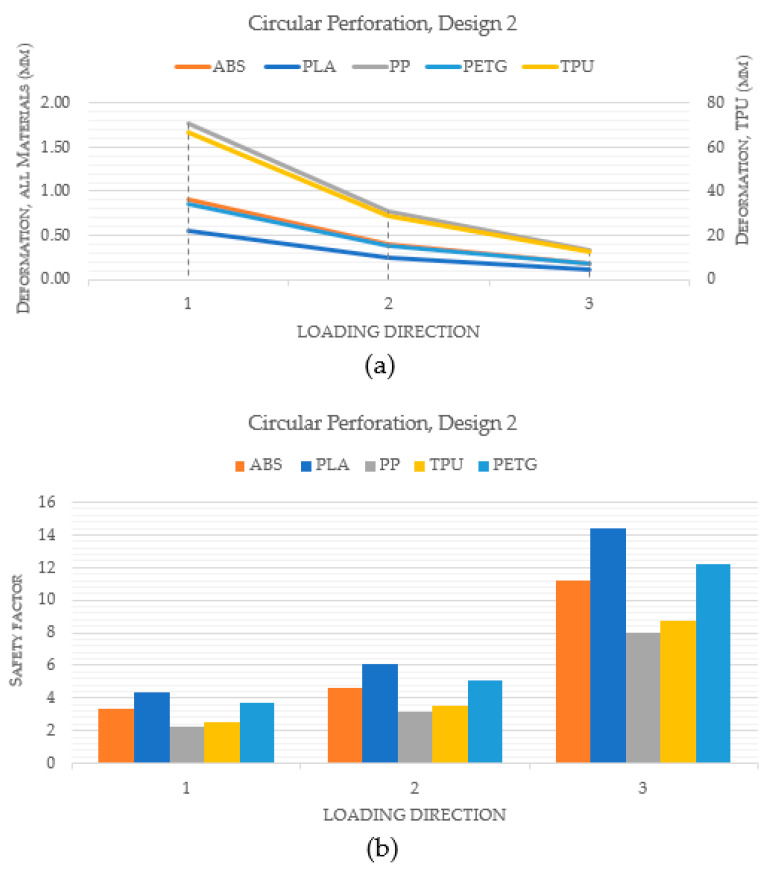
Comparison of different materials with circular perforations and Design 2 (**a**) Deformation; (**b**) Safety factor.

Figure 10 compares the performance of various materials for a hand splint with circular perforations and design 1, in which the holes are spaced equally apart by a linear pattern. Indeed, Figure 10a makes it clear that the PLA material exhibited the least deformation when compared to ABS, PP, TPU, and PETG. Additionally, it was noted that TPU deformed the most among the materials. Additionally, PLA had the highest safety factor across all loading directions, as shown in Figure 10b. This suggests that, in terms of stiffness and strength, PLA is the best material for hand splints. However, the materials were once more assessed in a similar way using design 2 where perforations are dispersed all over the hand splint, in order to further validate the results. Figure 11a demonstrates yet again that PLA had the smallest degree of deformation among all materials. Furthermore, Figure 11b illustrates that PLA had the greatest safety factor across all loading directions across all the materials.

**Figure 12:**
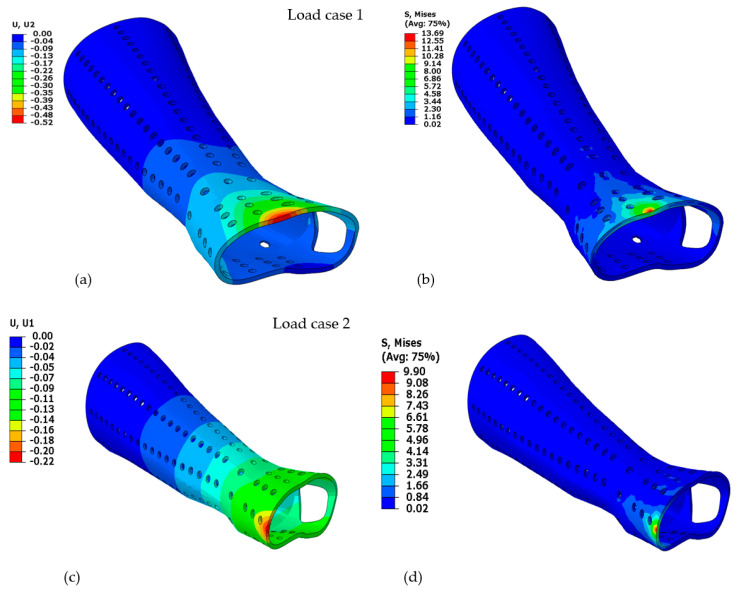

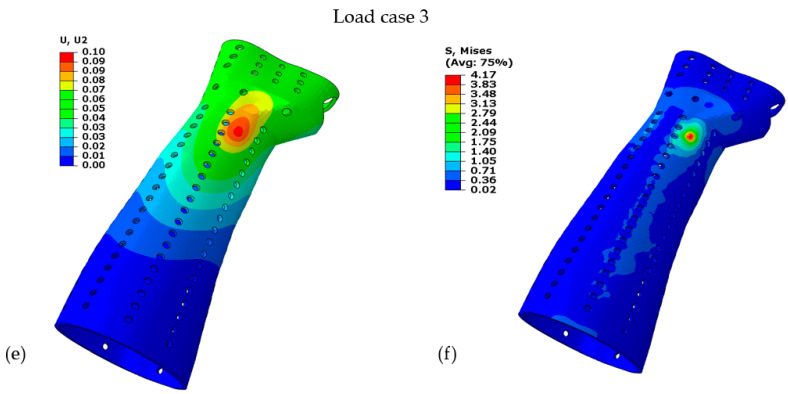
PLA 3 mm thick splint, circular perforation, Design 2 (**a**) Deformation under load direction 1; (**b**) Von Mises stress under load direction 1; (**c**) Deformation under load direction 2; (**d**) Von Mises stress under load direction 2; (**e**) Deformation under load direction 3; (**f**) Von Mises stress under load direction 3.

The second analysis compared various designs for PLA material that include different perforation shapes. The PLA material was chosen because the prior analysis determined that it was the most appropriate material. The PLA material had also been endorsed by numerous studies in the past for use in medical and other industrial products because of its superior characteristics and environmental friendliness [59,60,61,62]. Figure 12a–f displays the deformation and Von Mises stress for the PLA-based hand splint for Design 2 with circular perforations under three loading directions.

The performance index (*PI*) was computed for various designs with correspondingly variable perforation shapes. It is particularly important to use the *PI* to compare the performance of each design to the solid model, which is assumed to have the highest safety factor and least deformation. The Equation (1) was used to calculate the *PI*. It implies that a design’s stiffness and resilience are superior when *PI* is higher.

(1) PI=SFSFSUUS

where *U* is the deformation in the given design and corresponding perforation shape, *U_S_* is the deformation of the solid splint, *SF* is the safety factor in the given design and corresponding perforation shape, and *SF_S_* is the safety factor of the solid splint.

**Figure 13:**
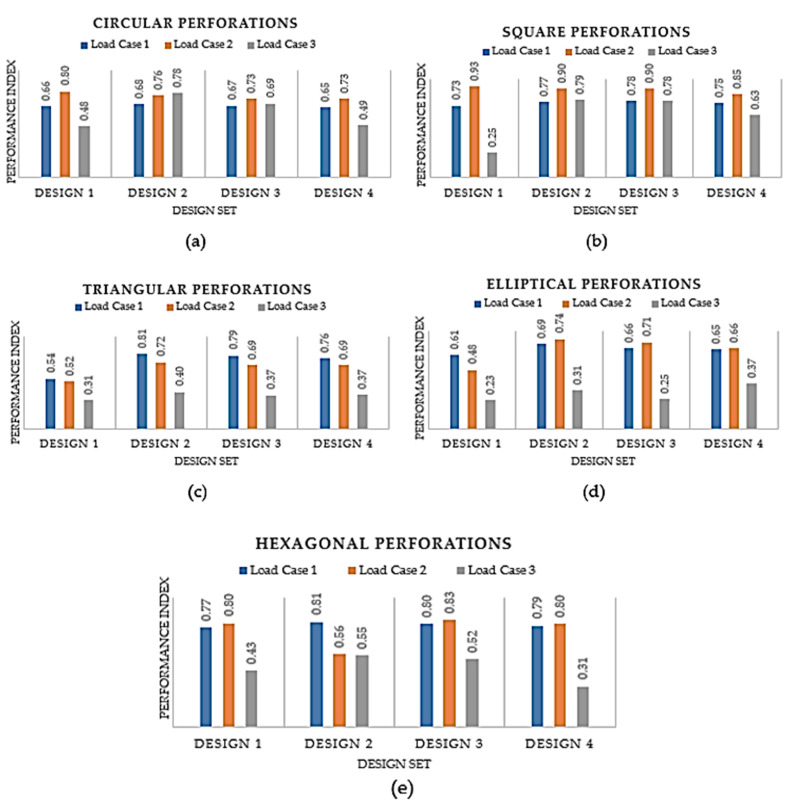
Performance Index of different designs corresponding to various perforation shapes (**a**) Circular; (**b**) Square; (**c**) Triangular; (**d**) Elliptical; (**e**) Hexagonal.

According to Figure 13, under all applied loading conditions, the *PI* is predominantly decreasing as the number of perforations increases from Design 2 to Design 4. It is evident since the material’s strength and stiffness diminish as the number of perforations grows. However, it is apparent that even if the *PI* is plunging for the three designs, the decline is not conspicuous and the designs do not fail. Out of Designs 2, 3, and 4, the one with the most perforations (Design 4) was favored since it would offer attractive looks and more airflow without drastically lowering its sturdiness and rigidity.

When Design 1 is compared to Designs 2, 3, and 4, it is obvious that the latter three are slightly more effective. It can be explained by the fact that the perforations in Design 1 were concentrated in the middle of the splint, but the perforations in Design 2 were dispersed all over the splint. However, it can be observed that Design 1 is superior in some circumstances. For instance, Design 1 is marginally better than Design 2 in perforations with circular, square, and hexagonal shapes. It can be explained by the fact that the perforations were spread throughout the splint in Design 2, so some perforations may have fallen in some intricate regions leading to higher stress concentration and thus higher deformation. Accordingly, Design 4 could be chosen based on this analysis because it offers increased ventilation without considerably reducing the splint’s strength. Additionally, it can be seen that all perforation shapes performed similarly, as illustrated in Figure 13, indicating that the influence of the perforation shapes is not particularly noticeable. However, out of all the perforation forms, square perforations appeared to have worked the best. Furthermore, it should be noted that the influence of perforation shapes can be more pronounced if their size is increased relative to the size that is considered in this work. 

**Figure 14:**
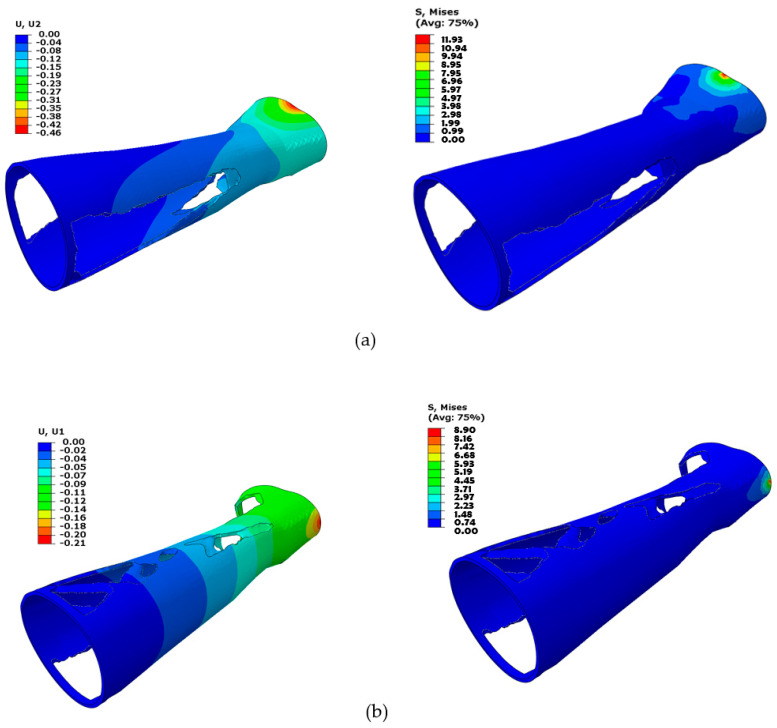

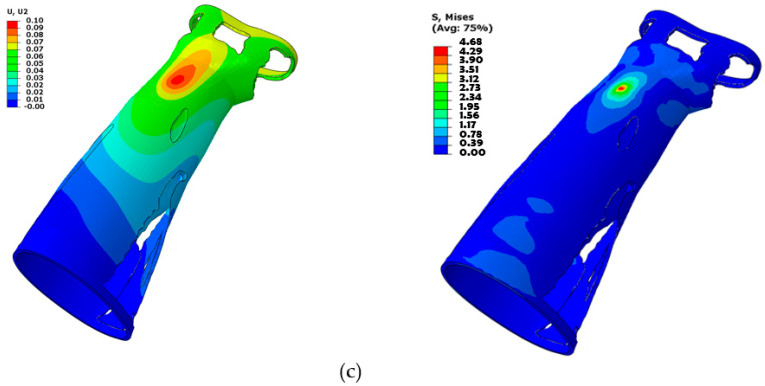
Analysis of topology-optimized splint structure for three load directions (**a**) Load case 1; (**b**) Load case 2; (**c**) Load case 3.

Along with the regular perforation shapes, the solid splint was also topology-optimized to remove unwanted material and provide the user with a lightweight and well-ventilated splint. Figure 14 displays the topology-optimized splint’s FE evaluation results for each of the three loading scenarios. It can be demonstrated that higher Von Mises stresses are attained at the location where the load is applied, and stresses decrease as one moves away from the area. As a result, elements close to the fixed boundary area (away from the wrist part) were deleted by the optimization solver because they were not carrying any substantial load and hence were not contributing to the splint’s stiffness. The optimization solver iterated until the maximum stiffness was reached with the applied constraints and specified maximum iterations.

**Figure 15:**
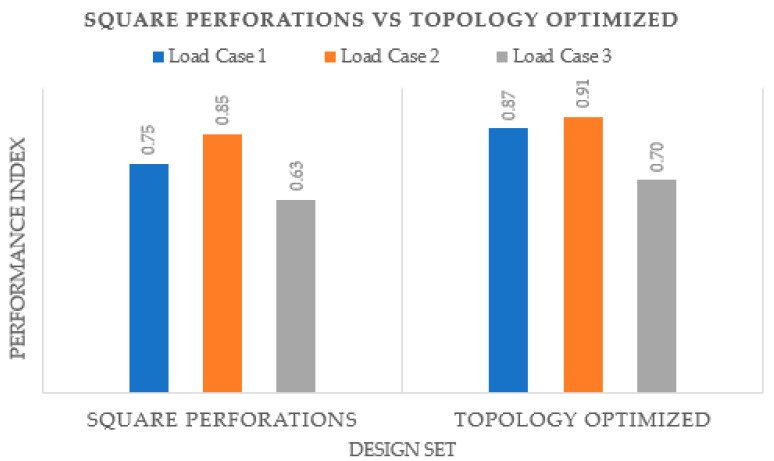
Comparison of hand splints with square perforations and topology optimized.

It is explicit that the topology-optimized hand splint as depicted in Figure 15 produced better results in terms of *PI*. It can be associated with the fact that the topology-optimized process uses an automated method in which the material is eliminated methodically so that the structure’s overall strength is not significantly impacted. This finding is consistent with that observed by Kumar and Chhabra [63]. Moreover, it addresses the vulnerable portions or locations that are susceptible to wearing and rupture by reinforcing them while producing a stronger and more durable product. It also lowers the chance of structural collapse and enhances safety by removing problematic segments and sites where stress can build up. On the other side, it is quite likely that the perforations in the standard-shaped perforated design may have landed on weak spots, thus weakening the design. Furthermore, the perforation patterns can lead to increased stress concentrations and subpar design performance. Topology optimization offers several advantages, but it also has many drawbacks. For instance, it offers a list of numerous designs, which frequently makes it difficult for the designer to choose the best one, thus requiring an additional step of further validating the suggested design. Topology optimization usually recommends designs that are challenging to manufacture, even using AM, and that require more time to prepare the part before producing it. The designs are frequently so intricate that mass production becomes a major problem. Additionally, there are expenses associated with developing software that is tailored for topology optimization, raising the price of topology-optimized solutions. It might be difficult to define constraints in topology optimization because too many constraints could undermine the resulting solution while fewer constraints lead to an enormous number of design variations, making it difficult for the designer to settle on one final design. Additionally, defining the ideal set of input parameters for topology optimization demands a great deal of knowledge and skills. As opposed to the topology-optimized structure, the splint with regular perforations has a relatively simpler design and is easier to fabricate. Therefore, both approaches have advantages and disadvantages, and the user must decide which approach to adopt based on the available resources and requirements.

**Figure 16:**
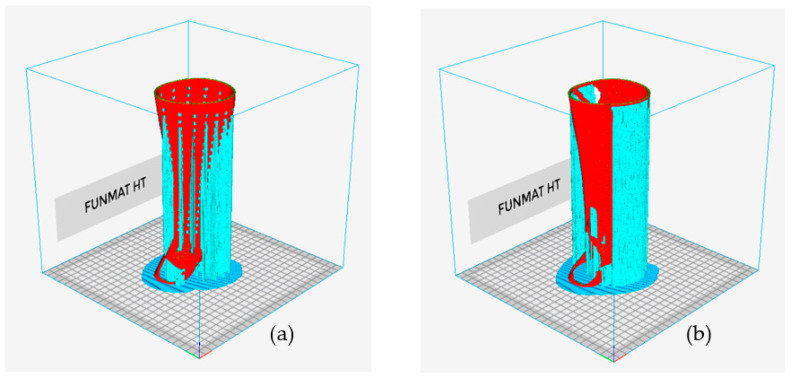
Support structure generated for hand splint with (**a**) square perforations and (**b**) topology optimization.

The next step was to create the two hand splints using FDM and discuss their manufacturability. The splint needs to be orientated properly for FDM manufacturing as a first step. Table 5 displays the different orientations of the various types of hand splints as well as the relevant performance metrics, such as material consumption and manufacturing time. Orientation 4 is the most suitable given that it resulted in the lowest material consumption and building time for the solid, perforated, and topology-optimized splints, respectively. In addition, it was discovered that the topology-optimized models had the lowest material consumption and build times when compared to the solid and the square-perforated models. This is because hand splints with square perforations have numerous square holes that need to be supported to prevent collapsing, as shown in Figure 16a. Additionally, hand splints with square holes contain an abundance of little surfaces that require support structures. The topology-optimized model, on the other hand, had a smaller volume of support material since it had a few large perforations and fewer surfaces (see Figure 16b). The material usage in square perforations, however, was also observed to be relatively high, particularly when compared to both solid and topology-optimized models. The cost savings associated with using less model material (square perforations) are therefore not possible because there is so much support material used in hand splints with square holes. The topology-optimized models, however, can reap the benefits of less material usage and lower 3D printing costs, which is the same as the conclusions stated in [30]. It is also important to note that the type and quantity of support structures employed can vary depending on the slicing program. Additionally, slicing software other than INTAMSUITE might yield different supports and volumes.

**Figure 17:**
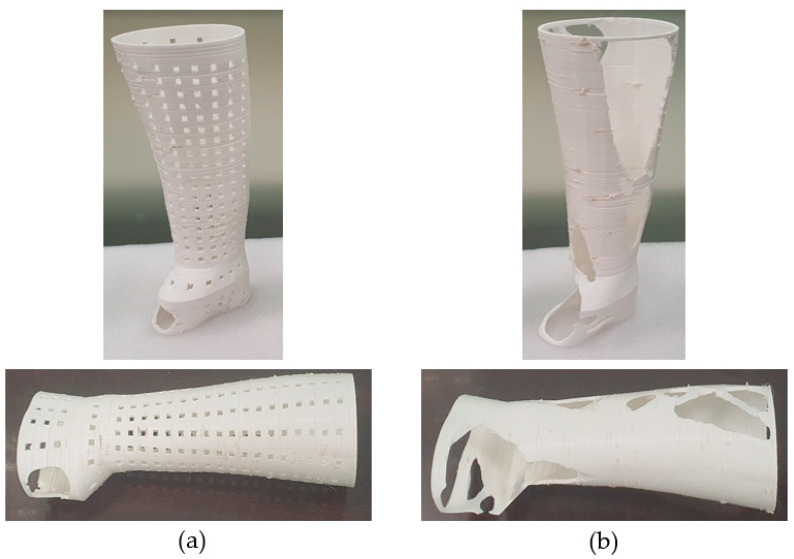
Custom-made 3D printed splint (**a**) square perforations (**b**) topology optimization.

The square-perforated hand splint required approximately 194 g of material (model plus supports) and took 34 h 23 min to print. The topology-optimized splint (option 2) consumed approximately 154 g of total material and needed 25 h and 21 min to print. Figure 17a,b demonstrate the completed 3D-printed hand splints with square perforations and topology optimization, respectively. The lack of symmetry in the splint design along the z-axis can be used to explain a too-large void on one side in the topology-optimized structure (Figure 17b). The top surface elements were closer to the load-bearing area, hence the optimization solution looked for (soft) low relative density elements that were more prevalent at the bottom than the top for elimination. As a result, more elements were omitted from the bottom surface because they do not add to the overall stiffness of the splint. The topology-optimized and square-perforated hand splints weighed about 104.9 g and 124.7 g, respectively. As before, it is demonstrated that the topology-optimized model weighs less than the square-perforated splint. It may be related to the fact that the topology-optimized procedure employed a software-driven mechanism in which the material is wiped prudently so that the structure’s strength-to-weight ratio is adequate. Additionally, it targeted the weak points or regions that are prone to deformation and breakage by strengthening them while delivering a more robust and long-lasting product.

It took 30 to 40 min to remove the supports for the topology-optimized hand splint, but the hand splint with square perforations required 150 to 170 min. The work of removing supports for square-perforated hand splints became rather difficult due to the omnipresent perforations and the time-consuming procedure of removing the little supports from every square perforation. The topology-optimized hand splint weighed about 26% less than the solid hand splint, while the square-perforated hand splint had 12% less weight than the solid splint.

## 4. Conclusions

This research established the most suitable design and material for an upper limb splint based on decreased Von Mises stresses and weight. A comparative examination of various materials and designs was carried out under various loading conditions. The material and design with the least deformation and the maximum safety factor were chosen for the hand splint. It was demonstrated that the PLA material deformed the least in comparison to ABS, PP, TPU, and PETG. Furthermore, PLA had the highest safety factor for all loading directions, indicating that PLA is the best material for hand splints. Of all the designs, the one with the most perforations that were evenly spaced across the splint is recommended because it offers appealing aesthetics and greater airflow without significantly lowering the splint’s durability and sturdiness. Additionally, the behavior of all perforation shapes appeared to be consistent, indicating that the impact of the perforation shapes is not very strong. However, among all the perforation shapes, square perforations showed to be the most effective. Furthermore, it was noted that, in terms of *PI*, the topology-optimized hand splint surpassed the hand splint with regularly shaped perforations. This may be attributed to the fact that the topology-optimized method employs an automated process in which the material is removed expertly such that the structure’s overall strength is not dramatically compromised. In addition, it improves safety and targets the weak spots that are sensitive to breaking and tear as well as reduces the likelihood of structural catastrophe by removing problematic sections and sites where stress might accumulate. Moreover, the hand splint with square perforations is reported to use more material than the topology-optimized splint, which increases production costs. Comparing the topology-optimized hand splint to the square-perforated hand splint, it required around 40 g less material overall and printed in about 9 h less time. When compared to a square-perforated hand splint, which had a final weight reduction of 12%, a topology-optimized variation had a weight reduction of about 26%. It is apparent that both approaches have benefits and drawbacks, therefore the user must select a strategy based on the resources and requirements that are accessible.

This work contributes by comparing various materials and offering a couple of techniques for perforating upper limb splints in addition to its design process and manufacturing feasibility using 3D printing. However, it has limitations in terms of the perforation size because it was not varied, and more research on dispersion patterns is recommended. Future studies should additionally vary the thickness of the hand splint, which was not undertaken in this work, to establish the impact of thickness on the perforated hand splints. It is also critical to assess the hand splint under more demanding loading conditions and with greater loads to corroborate the findings of this study. The fabricated splint must also be put to the test on a real patient as part of a clinical trial.

## Figures and Tables

**Table 1:** Description of Perforation shapes and distribution.

Perforation Shape	Design		Area per Perforation (mm^2^)
	Distribution/Pattern	Dimension (mm)	
Circular	Design 1: Linear/Packed; pitch—10 mm	Radius of the circular perforation—2.5	19.625
Square	Design 2: Round/Scattered; 45° (8)		
Triangular	Design 3: Round/Scattered; 30° (12)	Side of the square perforation—4.43	
Elliptical	Design 4: Round/Scattered; 24° (15)		
Hexagonal		Side of the equilateral triangle—6.732	
		
		Major axis of the ellipse—8	
		Minor axis of the ellipse—3.125	
		
		Side of the hexagon—2.7484	

**Table 2:** Properties of the 3D printing materials.

Material	Properties		
	Young’s Modulus (MPa)	Poisson Ratio	Yield Strength (MPa)
PLA	3466 [37]	0.30 [37]	60 [38,39]
ABS	2100 [40]	0.35 [40]	45 [41,42]
TPU	28.5 [43,44]	0.39	34 [43]
PP	1070 [45]	0.42 [45]	30 [46]
PETG	2200 [47,48]	0.33 [47]	50 [48,49,50]

**Table 3:** Process parameter settings.

Parameter	Set Value
Analysis type	Topology optimization (condition based)
Objective	Maximize stiffness (minimize compliance)
Initial mass target	70% of the design space volume
Element type	C3D4
Geometry constraints	Frozen Area (loads and boundary conditions)

**Table 4:** Process variables used in the FUNMAT HT 3D printer.

Description	3D Printer Settings [58]
Printing technology	FDM
Build platform	High borosilicate glass
Connectivity	SD card
Extruder nozzle diameter	0.4 mm
Layer thickness	0.1 mm
Print speed	60 mm/s
Extruder temperature	220 ℃
Chamber temperature	50 ℃
Platform temperature	50 ℃
Filament diameter	1.75 mm
Input file	STL
Infill density	100%

**Table 5:** Comparing the performance of various part orientations.

Design Set	Orientation	Material Consumption (Grams)	Build Time (Hours)	Build Cost (USD)
Square Perforations, Design 4	O1/0°	333	50.38	21.50
	O2/60°	262	51.08	16.93
	O3/75°	224	40.77	14.48
	O4/90°	194	34.38	12.54
Topology Optimization, Option 1	O1/0°	235	33.90	15.15
	O2/60°	180	28.78	11.64
	O3/75°	170	26.80	10.96
	O4/90°	158	24.63	10.19
Topology Optimization, Option 2	O1/0°	292	41.22	18.83
	O2/60°	171	28.52	11.04
	O3/75°	167	27.57	10.76
	O4/90°	154	25.35	9.94
Topology Optimization, Option 3	O1/0°	264	37.55	17.03
	O2/60°	163	27.07	10.50
	O3/75°	161	26.45	10.38
	O4/90°	158	25.25	10.17
Solid	O1/0°	330	45.97	21.31
	O2/60°	188	30.35	12.15
	O3/75°	182	30.12	11.72
	O4/90°	170	28.23	10.94

## Data Availability

The data presented in this study are available in the article.
